# Trends, Characteristics and Treatment Outcomes of Patients with Drug-Resistant Tuberculosis in Uzbekistan: 2013–2018

**DOI:** 10.3390/ijerph18094663

**Published:** 2021-04-27

**Authors:** Khasan Safaev, Nargiza Parpieva, Irina Liverko, Sharofiddin Yuldashev, Kostyantyn Dumchev, Jamshid Gadoev, Oleksandr Korotych, Anthony D. Harries

**Affiliations:** 1 Republican Specialized Scientific Practical Medical Centre of Phthisiology and Pulmonology under Ministry of Health of the Republic of Uzbekistan, 1 Majlisiy Str., Tashkent 100086, Uzbekistan; nargizaparpieva@gmail.com (N.P.); liverko@yandex.ru (I.L.); Sharaf.yuldashev@gmail.com (S.Y.); 2 The Charitable Organization “Ukrainian Institute on Public Health Policy”, 02000 Kyiv, Ukraine; dumchev@uiphp.org.ua; 3 World Health Organization Country Office Uzbekistan, 16, M.Tarobiy Str., Tashkent 100100, Uzbekistan; 4 World Health Organization Regional Office for Europe, UN City, Marmorvej 51, DK-2100 Copenhagen, Denmark; 5 International Union against Tuberculosis and Lung Disease, 68 Boulevard Saint Michel, 75006 Paris, France; adharries@theunion.org; 6 London School of Hygiene and Tropical Medicine, Keppel Street, London WC1E 7HT, UK

**Keywords:** Uzbekistan, Tashkent city, drug-resistant TB, multidrug-resistant TB, extensively drug-resistant TB, unfavorable treatment outcomes, death, loss to follow-up, operational research, SORT IT

## Abstract

Uzbekistan has a high burden of drug-resistant tuberculosis (TB). Although conventional treatment for multidrug-resistant TB (MDR-TB) and extensively drug-resistant TB (XDR-TB) has been available since 2013, there has been no systematic documentation about its use and effectiveness. We therefore documented at national level the trends, characteristics, and outcomes of patients with drug-resistant TB enrolled for treatment from 2013–2018 and assessed risk factors for unfavorable treatment outcomes (death, failure, loss to follow-up, treatment continuation, change to XDR-TB regimen) in patients treated in Tashkent city from 2016–2017. This was a cohort study using secondary aggregate and individual patient data. Between 2013 and 2018, MDR-TB numbers were stable between 2347 and 2653 per annum, while XDR-TB numbers increased from 33 to 433 per annum. At national level, treatment success (cured and treatment completed) for MDR-TB decreased annually from 63% to 57%, while treatment success for XDR-TB increased annually from 24% to 57%. On multivariable analysis, risk factors for unfavorable outcomes, death, and loss to follow-up in drug-resistant TB patients treated in Tashkent city included XDR-TB, male sex, increasing age, previous TB treatment, alcohol abuse, and associated comorbidities (cardiovascular and liver disease, diabetes, and HIV/AIDS). Reasons for these findings and programmatic implications are discussed.

## 1. Introduction

Drug-resistant tuberculosis (DR-TB) has become a major public health concern in many countries. Multidrug-resistant TB (MDR-TB—resistance to isoniazid and rifampicin) and extensively drug-resistant TB (XDR-TB—MDR-TB with added resistance to fluoroquinolones and second-line injectable drugs) are the most concerning types of drug-resistant TB because of difficulties with diagnosis and effective treatment [1].

The prevalence of MDR-TB and XDR-TB as a proportion of the number of TB cases has remained stable over the past several years [1], although in the World Health Organization’s (WHO) European Region, and especially in the countries of the former Soviet Union including Uzbekistan, it has been increasing [2]. The diagnosis of MDR-TB has improved with the advent and scale-up of the automated molecular diagnostic assay Xpert MTB/RIF, which enables a rapid diagnosis of both TB and rifampicin resistance within two hours [3]. Most rifampicin-resistant TB (RR-TB) diagnosed by Xpert MTB/RIF is accompanied by isoniazid resistance, and therefore RR-TB is routinely treated as MDR-TB [1].

The diagnosis of XDR-TB has also improved in recent years with the advent of second line probe assays (LPA-MTBDR*sl*). Traditionally, the identification of first- and second-line drug resistance required mycobacterial culture and conventional phenotypic drug susceptibility testing (CDST) using solid or liquid culture media, a procedure that takes several weeks. Line probe assays (LPAs), which use nucleic acid amplification and gene mutation detection, are an alternative and much faster molecular diagnostic method, taking between 2–3 days [4]. LPAs are recommended by WHO for diagnosing first-line drug resistance to rifampicin and isoniazid [5], and for detecting second-line drug resistance in people with a prior diagnosis of rifampicin-resistant TB or MDR-TB [6].

In 2019, an estimated 465,000 persons globally had MDR/RR-TB, of whom 206,030 (44%) were diagnosed and notified, a 10% increase on the previous year [1]. Of these persons with MDR/RR-TB, 6% had XDR-TB. The number of persons with MDR/RR-TB enrolled on treatment in 2019 was 177,099 (86% of notified cases) [1]. Traditionally, treatment of MDR/RR-TB requires standardized “conventional” treatment regimens for up to 24 months with second-line anti-TB drugs, which are less effective, more expensive and associated with more adverse drug events compared with first-line drugs. Under programmatic conditions, successful treatment outcomes with these conventional regimens have been achieved in only half of all patients, with treatment success reaching 57% in the most recently reported 2017 cohort [1]. The development and use of shorter MDR-TB treatment regimens, however, has given grounds for optimism. Under operational research conditions in Asia and Africa, short course regimens ≤ 12 months have been associated with successful treatment outcomes > 75% with low relapse rates [7,8]. These findings have been confirmed recently by a randomized controlled trial [9]. The World Health Organization (WHO) has now recommended a short course regimen of 9–12 months for MDR-TB, provided certain conditions are met [10], and because of ototoxicity from second-line injectable agents [11] these are also being phased out. WHO has further recommended that fully oral short course regimens be considered in the future [12].

In the last two decades, Uzbekistan has seen a significant decrease in the number of reported TB cases from 94 per 100,000 people in 2003 to 49 per 100,000 in 2019 [1]. Despite this progress, the country still grapples with a high burden of DR-TB. A drug resistance survey in 2010–2011 found a prevalence of MDR-TB of 23% in new patients and 62% in previously treated patients [13]. The country is currently regarded as one of 30 countries in the world with a high MDR-TB burden [14].

Standardized “conventional” MDR-TB treatment has been available countrywide since 2013, being first started in the Republic of Karakalpakstan in 2003 with support from Medecins Sans Frontieres (MSF). MSF has supported MDR-TB treatment optimization by piloting a shorter 9–11-month regimen since 2013 in the Republic of Karakalpakstan, and shorter MDR-TB treatment regimens are also being piloted in Tashkent city and Samarkand oblast since June 2018. Conventional individualized XDR-TB treatment has been available for the whole country since 2015.

Uzbekistan is now engaged with the WHO and other countries in piloting and scaling-up modified fully oral shorter treatment regimens for MDR/RR-TB patients from 2020 under operational research conditions. To help with the strategic direction of this initiative, the National TB Program (NTP) wanted background information about trends of DR-TB and treatment outcomes over the last 5–6 years as well as specific risk factors for unfavorable outcomes, death, and loss to follow-up in patients with MDR/RR-TB and XDR-TB treated with the established “conventional” regimens in the country. This led to the implementation of the current study.

The aim of this study was to document the trends, characteristics, and treatment outcomes of patients with DR-TB enrolled for treatment in Uzbekistan between 2013 and 2018. Specific objectives were to determine: (1) trends in annual numbers of patients with different types of DR-TB who were enrolled in treatment; (2) demographic and clinical characteristics of patients with MDR/RR-TB and XDR-TB enrolled in treatment; (3) trends in treatment outcomes for MDR/RR-TB and XDR-TB patients enrolled in treatment between 2013 and 2017; and (4) in Tashkent city, risk factors for unfavorable outcomes, death, and loss to follow-up in patients with MDR/RR-TB and XDR-TB enrolled on treatment in 2016–2017.

## 2. Materials and Methods

### 2.1. Study Design

This was a descriptive study using secondary aggregate data for objectives 1–3 and a cohort study using secondary individual patient data for objective 4.

### 2.2. Setting

#### 2.2.1. General Setting

Uzbekistan is situated in central Asia with an estimated population of 32,656,660 in 2018 [15]. The country is administratively divided into 12 regions, the city of Tashkent and the Republic of Karakalpakstan. In the past two decades, there have been major reforms of the Uzbek health system including all levels of care, as well as financing and governance. To ensure sustainable improvements in the quality of care, the health system makes ongoing efforts to update treatment protocols, revise medical education, provide continuous professional development, and implement quality assurance and improvement frameworks [16].

#### 2.2.2. TB Control

The national effort in fighting TB is chaired by the Republican Specialized Scientific Practical Medical Centre of Phthisiology and Pulmonology under Ministry of Health of the Republic of Uzbekistan. There is an NTP that embraces a network of Regional centers of phthisiology and pulmonology, city TB dispensaries, and district TB departments. The facilities have 15 MDR-TB and 6 XDR-TB wards. TB patients receive standardized and controlled treatment in accordance with national guidelines that over the study period have adhered to WHO guidelines [17,18,19,20,21]. TB diagnosis and treatment is free of charge for all patients.

#### 2.2.3. TB Diagnostics

The laboratory network consists of two national reference laboratories, six regional bacteriological laboratories, and district microscopy and GeneXpert laboratories. Microscopy laboratories are also available at primary health care centers. Each regional laboratory is equipped with modern systems for TB diagnosis: Xpert MTB/RIF (Cepheid Inc, Sunnyvale, CA, USA), LPAs (Hain LifeScience GbH, Nehren, Germany) and mycobacterial culture and phenotypic drug susceptibility testing using BACTEC MGIT (Becton Dickinson and Company, NJ, USA). As of 2020, the country has a total of 67 GeneXpert MTB/RIF instruments, 17 BACTEC MGIT machines, and 13 LPA-HAIN instruments.

#### 2.2.4. TB Treatment and TB Treatment Outcomes

TB treatment regimens are in line with regimens recommended by the WHO [17,18,19,20,21], and these are shown in Table 1. TB treatment outcomes are also standardized [22], although adapted to Uzbekistan.

#### 2.2.5. TB Surveillance, Monitoring, and Evaluation

The NTP uses a paper-based TB surveillance system, backed up by several electronic databases (including laboratory databases and databases for MDR-TB patients). These databases are regularly updated by monitoring and evaluation officers in the regions and results sent upwards to the national level. National TB reporting is based on the paper-based surveillance data.

### 2.3. Study Population

For objective 1–3, the study population was the aggregate numbers of TB patients enrolled for treatment in Uzbekistan between 2013 and 2018, and for objective 4, the study population was the individual number of patients with MDR/RR-TB and XDR-TB enrolled for treatment in Tashkent city in 2016–2017.

### 2.4. Data Variables, Data Collection, and Sources of Data

For the first three study objectives dealing with aggregate data, the variables included: year; total TB; total drug-susceptible TB; total poly drug resistant TB; total RR/MDR-TB; total XDR-TB; sex; age group; region; TB localization; category of TB; enrolled for MDR/RR-TB and XDR-TB treatment; and treatment outcomes according to the Uzbekistan NTP that included treatment success (cured plus treatment completed), failure, death, loss to follow-up, treatment continues up to and after surgery, and change to XDR-TB treatment because of initial misclassification. For the fourth objective dealing with individualized data, the variables included: year; enrolled for MDR/RR-TB treatment and XDR-TB treatment; all treatment outcomes as mentioned above in objective 3; sex; age group; TB localization; category of TB; co-morbidities; and socio-economic characteristics as recorded in the patient treatment cards.

The source of data was the national Excel-based data base at the NTP. Depersonalized data was extracted for all objectives into a separate Excel spread sheet between August and December 2020. For objective 4, individual patient data was collected from treatment cards by the health-care workers with access to the data. The data was de-identified and entered into an MS Excel spread sheet and cross-checked.

### 2.5. Analysis and Statistics

For aggregate data, a descriptive analysis was performed using frequencies and proportions. Frequencies of each treatment outcome were disaggregated by all variables. The three main outcomes of interest were unfavorable treatment outcome (anything other than treatment success), death, and loss to follow-up. In univariable analysis, risk ratios (RR) with 95% confidence intervals were estimated using Poisson regression with robust standard errors. Variables with *p*-values < 0.1 in univariable analysis were included into multivariable Poisson regression models, producing adjusted RRs. Analysis was done using R software version 4.0.3 (R Foundation for Statistical Computing, Vienna, Austria). Levels of significance were set at 5% (*p* < 0.05).

## 3. Results

### 3.1. Trends in Annual Numbers of Patients with Different Types of Drug-Resistant TB Enrolled for Treatment

**Figure 1:**
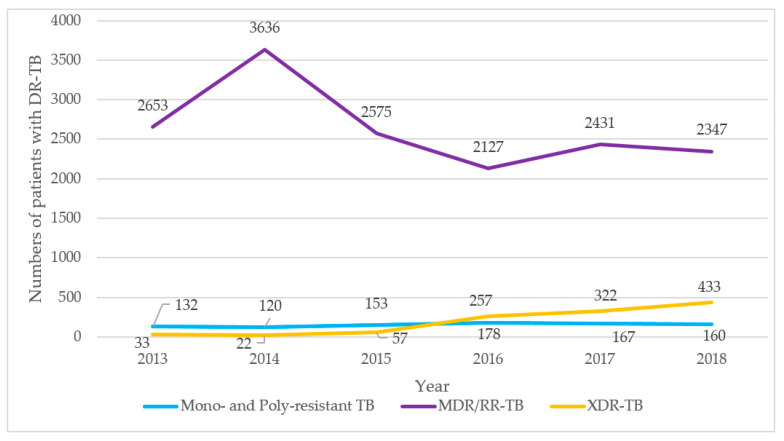
Trends in numbers of patients with different types of drug-resistant tuberculosis enrolled for treatment in Uzbekistan between 2013 and 2018. TB = tuberculosis; DR-TB = drug resistant TB; MDR-TB = multidrug-resistant TB; RR-TB = rifampicin-resistant TB; XDR-TB = extensively drug resistant TB.

Trends in numbers of patients with mono- and poly drug resistant TB, MDR/RR-TB, and XDR-TB enrolled for treatment between 2013 and 2018 are shown in Figure 1. There were small numbers with mono- and poly drug resistant TB that varied from a minimum of 120 to a maximum of 178. There was an initial increase in MDR/RR-TB in the first two years, and thereafter numbers declined and were relatively stable between 2127 to 2575 per year (this constituted 11% to 12% of all TB patients enrolled for treatment). The number of XDR-TB patients was initially less than 50 per year, but thereafter numbers increased to 4433 (2% of all TB patients) in 2018.

### 3.2. Demographic and Clinical Characteristics of Patients Enrolled for MDR-TB and XDR-TB Treatment

Demographic and clinical characteristics of patients enrolled for MDR-TB and XDR-TB treatment are shown in Table 2. For both MDR-TB and XDR-TB there were nearly twice as many males as females and the commonest age groups were 30–44 and 45–64 years, respectively. In terms of regions, the Republic of Karakalpakstan had the highest numbers with MDR-TB and XDR-TB, with other affected oblasts being Tashkent city and Tashkent oblast, Fergana oblast, Andijan oblast, and Samarqand oblast. There was a sizeable number of patients in the prison situated in Tashkent city. The most common type of TB was pulmonary TB. For MDR-TB, the three most common categories of TB were new, relapse, and treatment after failure, while for XDR-TB, the most common categories were treatment after failure, new, and relapse.

### 3.3. Trends in Treatment Outcomes of MDR/RR-TB and XDR-TB Patients Enrolled in Treatment

**Figure 2:**
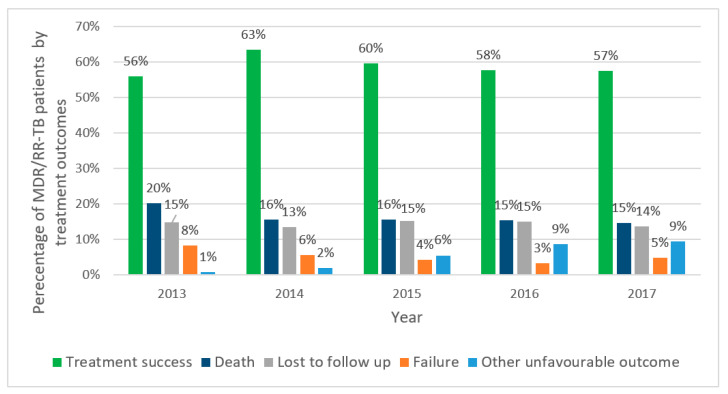
Treatment outcomes of MDR/RR-TB patients enrolled in MDR-TB treatment in Uzbekistan between 2013 and 2017. TB = tuberculosis; MDR-TB = multidrug-resistant TB; RR-TB = rifampicin resistant TB; other unfavorable outcome—continues on treatment up to and after surgery or changed to XDR-TB treatment.

Treatment outcomes for MDR/RR-TB patients enrolled in treatment are shown in Figure 2. Treatment success initially increased from 56% to 63%, and thereafter slightly declined over the next three years to 60% and then down to 57%. The proportion of patients who died was 20% in 2013, and this decreased and stabilized at about 15% thereafter. Loss to follow-up was fairly similar each year, varying from 13% to 15%. Failure remained at 5% or below. There was a gradual increase in other unfavorable outcomes from 1% to 9%, mainly due to an increase in the number of patients changing to XDR-TB treatment regimens as a result of initial misclassification.

**Figure 3:**
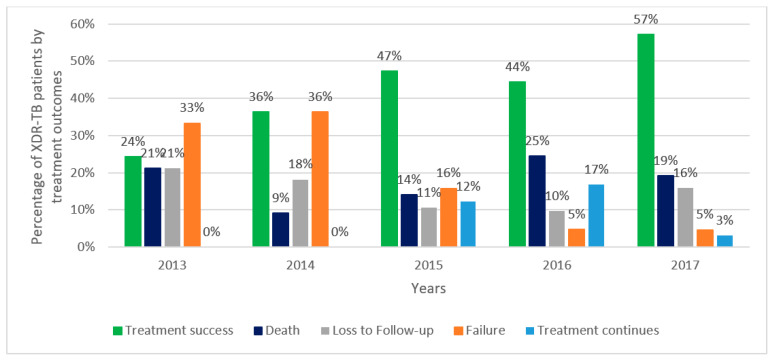
Treatment outcomes of XDR-TB patients enrolled in XDR-TB treatment in Uzbekistan between 2013 and 2017. TB = tuberculosis; XDR-TB = extensively drug resistant TB; Treatment continues = treatment continues up to and after surgery.

Treatment outcomes for XDR-TB patients enrolled on treatment are shown in Figure 3. Treatment success was initially low at 24%, but this gradually increased to reach 57% in 2017. Death varied from year to year from a low of 9% to a high of 25%, and this was similar to loss-to-follow-up that varied from 10% to 21%. Failure was high in the first two years at 33% and 36%, respectively, and thereafter declined to a low of 5%. In 2015 and 2016, 12% and 17% of patients, respectively, continued treatment up to and after surgery, but this declined to 3% in 2017.

### 3.4. Risk Factors for Unfavourable Outcomes, Death, and Loss to Follow-Up in MDR/RR-TB and XDR-TB Patients Enrolled in Treatment in Tashkent City from 2016 to 2017

Risk factors for unfavorable treatment outcomes, death, and loss to follow-up are shown in Table 3, Table 4 and Table 5, respectively. On adjusted analysis, unfavorable treatment outcomes were significantly higher in patients with XDR-TB, patients with increasing age, those previously treated for TB, those who had concurrent liver disease or diabetes mellitus, and those who were reported to use alcohol (Table 3); death was significantly higher in patients with XDR-TB, patients with increasing age, those previously treated for TB, those who had concurrent cardiovascular disease, and those with HIV infection/AIDS (Table 4); loss to follow-up was significantly higher in patients enrolled in treatment in 2017 compared with 2016, patients with XDR-TB, and patients who were reported to use alcohol (Table 5).

## 4. Discussion

This is the first study in Uzbekistan to assess (i) at the national level using aggregate data, the trends, characteristics, and outcomes of patients with DR-TB enrolled for treatment over a six-year period (2013–2018) and (ii) in the city of Tashkent, using individual data, the risk factors for unfavorable treatment outcomes, death, and loss to follow-up in patients enrolled for MDR-TB and XDR-TB treatment over a two year period (2016–2017). There were three key findings.

First, over the six years, the registered numbers with mono-resistant and poly-resistant TB remained generally low and fairly stable, while numbers with MDR-TB initially increased and then stabilized. In contrast, numbers with XDR-TB increased particularly between 2015 and 2018. The low numbers reported with mono- and poly-resistant TB may have been due to poor coverage of drug-susceptibility testing for these first-line drugs. The increase in XDR-TB is most probably a reflection of the country introducing LPAs for second-line drug resistance from 2016, allowing wider national coverage for the bacteriological diagnosis of XDR-TB. Treatment for XDR-TB also became more available, which allowed the backlog of diagnosed patients to be treated. These findings are in line with WHO Global TB Reports over the last decade [1] and with previous studies from Central Asia [23].

The demographic and clinical characteristics of patients cumulatively enrolled in MDR-TB treatment over the six years were similar to those on XDR-TB treatment. These characteristics align with several recent reviews of DR-TB patients [24,25]. Twice as many males as females were enrolled. This is probably because males engage more in high risk behaviors such as smoking and alcohol abuse that put them at risk of TB [26], and they are also more likely to be migrants, homeless, or incarcerated, or inject drugs, all of which also increase their risk of TB and DR-TB [26,27,28,29]. In contrast, females in central Asia tend to stay at home and are less exposed to the risk of TB.

Second, there was a gradual decrease in treatment success in patients enrolled in MDR-TB treatment, which is in contrast to the WHO Global TB Reports, which have shown that treatment success worldwide has improved year on year from 50% in 2012 to 57% in 2017 [1]. In our cohorts, approximately one third of patients died or were lost to follow-up, with these two adverse outcomes staying relatively stable over time. However, the proportion of patients who continued treatment up to and after surgery and the proportion who changed to XDR-TB treatment (as a result of improved diagnostic capacity) both increased, and these were partly responsible for the declining treatment success. Lack of pharmacovigilance and high toxicity of some of the drugs may also have been responsible. In contrast, treatment success for XDR-TB more than doubled during the six years, and for the first time in 2017 surpassed 50%. This was mainly due to two factors: a marked decline in treatment failure and a decline in patients continuing up to and after surgery, both probably as a result of early diagnosis of second-line drug resistance and possible use of new drugs and new models of care.

Third, the independent risk factor analysis on treatment outcomes showed familiar findings. Unfavorable outcomes, death, and loss to follow-up were all significantly higher in XDR-TB compared with MDR-TB, in line with Global TB Reports and recent reviews that include previous studies from Uzbekistan [1,24,30,31,32]. Unfavorable outcomes and death were both associated with increasing age and previous TB treatment in line with previous studies [25,33]. However, there were variable findings with respect to comorbidities in that diabetes mellitus and liver disease were associated with unfavorable outcomes, while death was associated with HIV/AIDS and cardiovascular disease. Diabetes mellitus is associated with an increased risk of MDR-TB [34] and unfavorable treatment outcomes that include death and treatment failure [35]. Chronic liver disease has also been reported to be associated with unfavorable treatment outcomes [36]. The association between HIV infection and death in MDR-TB is well established [33,37], and mortality remains high even with the use of antiretroviral therapy (ART), probably because of delays in starting ART and increasing world-wide drug resistance to antiretroviral drugs. The association of death with cardiovascular disease is an interesting finding. Persons treated for TB have significantly higher long-term all-cause mortality compared with the general population, with most of these deaths being attributable to cardiovascular disease [38]. This association requires further and more detailed study. Lost to follow-up was higher in 2017 compared with 2016. Previous studies on patients lost to follow-up from Tashkent city have pointed to the long duration of hospitalization and poor communication between health care staff and TB patients as key contributing factors [39], but whether these played a part in our study is unclear. There was a strong association between loss to follow-up and alcohol abuse, and this association is well established [33].

The strengths of this study were the full national sample for aggregate data analysis and the large number of patients enrolled for MDR-TB and XDR-TB in Tashkent city for individual data analysis, which made it possible to assess independent risk factors. The conduct and reporting of the study was in line with the Strengthening the Reporting of Observational Studies in Epidemiology (STROBE) Guidelines [40].

There were, however, a number of limitations. The national aggregate data were obtained from the routine recording and reporting systems, and there may have been errors that we were unable to identify. As previously stated, the low numbers reported with mono- and poly-resistant TB may have been due to poor coverage of drug-susceptibility testing for these first-line drugs. The treatment regimens were not all the same during the six year period, and in the Republic of Karakalpakstan, shorter regimens have already been piloted since 2013 by MSF [41,42]. The selection of Tashkent city for the risk factor analysis means we cannot be sure about national representativeness of the findings. While we obtained data on many socio-demographic and clinical characteristics that affect treatment outcomes, they were not fully comprehensive and did not include information about the extent of parenchymal lung damage, co-morbidities such as chronic renal disease, and adjunctive treatments that might reduce treatment success [33]. We lacked detailed information about how the diagnoses of comorbidities such as diabetes and cardiovascular disease were made and what treatment was administered. There was a high HIV-prevalence amongst those tested, and this may have been due to selective referral of HIV-infected DR-TB patients to Tashkent city. We did not collect pharmacovigilance data during the study, and this prevented us from reporting on and analyzing any adverse drug events that might have contributed to poor treatment outcomes. Finally, we placed the outcome “treatment continues up to and after surgery” as unfavorable. This may be queried by some, as this outcome is not included in the WHO list of standardized outcomes [22]. However, it cannot be regarded as medication treatment success because of the need for surgical intervention, and therefore has to be included as “unfavorable”.

Despite these limitations, there are three important programmatic implications. First, whatever the reasons for the decrease in treatment success for MDR-TB, our findings point to the need for better MDR-TB treatment. As previously mentioned, short course regimens for MDR-TB have been shown in observational and randomized controlled studies to be safe and effective with treatment success >75% [7,8,9]. WHO recommends short course treatment provided patients have not been treated with second-line drugs or have had second-line drug resistance excluded [10]. WHO indeed suggests that countries consider fully oral short course regimens for the future [12]. The findings in this study from the last six years and a recent Markov modelling study showing cost-effectiveness of a fully oral bedaquiline-containing short course regimen [43] endorse this approach for MDR-TB in Uzbekistan. Pilot studies are already being developed and are underway.

Second, despite increasingly better treatment success with XDR-TB, the growing numbers of patients with this disease also point to the need for better treatment. The 90% favorable outcomes among patients with highly drug-resistant TB treated for six months with a combination of bedaquiline, pretomanid, and linezolid (BPaL) [44] are encouraging and pave the way for better and shorter treatment for XDR-TB. This new and emerging evidence has led WHO to update DR-TB treatment guidelines and provide specific advice on how to use shorter all-oral bedaquiline-containing regimens and BPaL regimens under operational research conditions [45]. These exciting treatment initiatives need to be accompanied by good clinical practice and pharmacovigilance.

Third, given the independent associations between various co-morbidities and unfavorable treatment outcomes in patients with MDR-TB and XDR-TB, it will be important to collect information on these at the start of treatment and act appropriately. For example, providing timely ART for HIV-infected patients, improving blood glucose control for those with diabetes, and giving direct-acting antiviral therapy to patients with hepatitis C-induced chronic liver disease can improve treatment outcomes and save lives [37,46,47].

## 5. Conclusions

This study showed that between 2013 and 2018, there was a stable number of MDR-TB patients enrolled for treatment at the national level in Uzbekistan, while numbers with XDR-TB increased. Treatment success in MDR-TB at the national level gradually decreased to below 60%, while treatment success in XDR-TB increased to above 50% in the 2017 cohort. In a risk factor analysis in patients enrolled to MDR-TB and XDR-TB treatment in Tashkent city, important socio-demographic and comorbidities were identified as increasing the risk of unfavorable outcomes, death, and loss to follow-up. Reasons for these findings and programmatic implications are discussed.

## Figures and Tables

**Table 1:** TB treatment regimens used in Uzbekistan.

Type of TB	Category	Previous Guidelines	New Guidelines
DS-TB	New	2HRZE/4HR	2HRZE/4HR
	Previously treated	3HRZE/5HRE	2HRZE/4HR
MonoDR-TB	New	3R-Z-E-Km-Lfx/6R-Z-E-Lfx	6R-Z-E-Lfx
	Previously treated	3R-Z-E-Km-Lfx/6R-Z-E-Lfx	9R-Z-E-Lfx-Lzd
PDR-TB	HE-resistance	3R-Z-E-Km-Lfx/6R-Z-E-Lfx	3Am-R-Z-Lfx-Lzd/9R-Z-Lfx-Lzd
	HZ-resistance	3R-Z-E-Km-Lfx/6R-Z-E-Lfx	3Am-R-E-Lfx-Lzd/9R-E-Lfx-Lzd
	HZE-resistance	3R-Z-E-Km-Lfx/6R-Z-E-Lfx	MDR-TB treatment
MDR/RR-TB	Short course regimen	4-6Km-Mfx-Cfz-Pto-Z-E-H^H^/ 5Mfx-Cfz-Pto-Z-E	4-6Km-Mfx-Cfz-Pto-Z-E-H^H^/ 5Mfx-Cfz-Pto-Z-E
	Standard long Regimen	8Km/Cm-Lfx-CS/PAS-Pto-Z-E/ 16Lfx-Cs/PAS-Pto-Z-E	Middle risk group 20Bdq-Lfx-Lzd-Cfz-Cs High risk group 20Bdq-Dlm-Lzd-Cfz-Cs High risk group According to DST, individual
Pre-XDR-TB	Resistance to FQ	8Km/Cm-6Bdq-Lzd-Cfz-Z-Pto-Cs/ 16Lzd-Cfz-Z-Pto-Cs	
	Resistance to SLI	6Bdq-Mfx-Lzd-Cfz-Z-Pto-Cs/ 18Mfx-Lzd-Cfz-Z-Pto-Cs	
XDR-TB	All	12Bdq-Lzd-Cfz-Imp/cln-Amx/clv-Pto-Cs/12Mfx-Lzd-Cfz-Pto-Cs	

DS-TB = drug-susceptible TB; MonoDR-TB = isoniazid mono-resistance; PDR-TB = polydrug-resistant TB which is resistant first line drugs other than rifampicin; MDR/RR-TB = multidrug resistant TB, resistant to isoniazid and rifampicin and rifampicin resistant TB; pre-XDR-TB = MDR-TB with either resistance to fluoroquinolones or injectable aminoglycosides/capreomycin; XDR-TB = MDR-TB with resistance to fluoroquinolones and injectable aminoglycosides/capreomycin. Several treatment options for MDR/RR-TB and pre-XDR-TB are being tested in Uzbekistan under operational research conditions. The numbers preceding the treatment regimen indicates the number of months of treatment. Middle and high risk groups indicate level of risk of treatment failure. H = isoniazid; H^H^ = high dose isoniazid; R = rifampicin; Z = pyrazinamide; E = ethambutol; Am = amikacin; Km = kanamycin; Lfx = levofloxacin; Lzd = linezolid; Mfx = moxifloxacin; Cfz = clofazamine; Pto = prothionamide; CS = cycloserine; PAS = para-amino salicylic acid; Bdq = bedaquiline; Imp/cln = imipenem-cilastatin; Amx/clv = amoxicillin-clavulanic acid; dlm = delaminid; FQ = fluoroquinolones; SLI = Second-line injectables.

**Table 2:** Demographic and clinical characteristics of patients enrolled for MDR-TB and XDR-TB treatment in Uzbekistan between 2013–2018.

Demographic and Clinical Characteristics		MDR/RR-TB		XDR-TB	
		n	(%)	n	(%)
Total		15769	(100.0)	1124	(100.0)
Sex	Male	10095	(64.0)	701	(62.4)
	Female	5674	(36.0)	423	(37.6)
Age group in years	0–14	132	(0.9)	29	(2.6)
	15–29	3977	(25.2)	294	(26.2)
	30–44	5192	(32.9)	368	(32.7)
	45–64	5216	(33.1)	376	(33.5)
	≥65	1252	(7.9)	57	(5.0)
Regions and prisons	Republic of Karakalpakstan	3682	(23.4)	294	(26.2)
	Tashkent city	1500	(9.5)	157	(14.0)
	Andijan oblast	1072	(6.8)	54	(4.8)
	Bukhara oblast	406	(2.6)	24	(2.1)
	Djizak oblast	559	(3.5)	39	(3.5)
	Kashkadarya oblast	786	(5.0)	15	(1.3)
	Navoi oblast	289	(1.8)	17	(1.5)
	Namangan oblast	867	(5.5)	33	(2.9)
	Samarqand oblast	1169	(7.4)	49	(4.4)
	Surkhandarya oblast	584	(3.7)	27	(2.4)
	Sirdarya oblast	402	(2.5)	29	(2.6)
	Tashkent oblast	1517	(9.6)	100	(8.9)
	Fergana oblast	1557	(9.9)	132	(11.7)
	Khorezm oblast	648	(4.1)	125	(11.1)
	Prison	731	(4.7)	29	(2.6)
Type of TB	Pulmonary	15396	(97.6)	1104	(98.2)
	Extrapulmonary	373	(2.4)	20	(1.8)
Category of TB	New	5004	(31.7)	173	(15.4)
	Relapse	4028	(25.5)	115	(10.2)
	Treatment after failure	3556	(22.6)	760	(67.6)
	Treatment after LTFU	335	(2.1)	44	(3.9)
	Retreatment other	2846	(18.1)	32	(2.9)

TB = tuberculosis; MDR-TB = multidrug-resistant TB; XDR-TB = extensively drug-resistant TB; LTFU = lost to follow up.

**Table 3:** Risk factors for unfavorable treatment outcomes in patients with TB enrolled in MDR-TB and XDR-TB treatment in Tashkent city, Uzbekistan, 2016–2017.

Variables		Enrolled to Treatment	Unfavorable Outcome		RR (95% CI)	*p-*Value	aRR (95% CI)	*p*-Value
		n	n	(%)				
Total		545	242	(44.4)				
TB dispensary	City Tashkent	124	56	(45.2)	Ref			
	Dispensary 1	44	21	(47.7)	1.1 [0.7–1.5]	0.77		
	Dispensary 2	87	42	(48.3)	1.1 [0.8–1.4]	0.65		
	Dispensary 3	85	40	(47.1)	1.0 [0.8–1.4]	0.79		
	Dispensary 4	57	22	(38.6)	0.9 [0.6–1.3]	0.42		
	Dispensary 5	148	61	(41.2)	0.9 [0.7–1.2]	0.51		
Year started treatment	2016	260	106	(40.8)	Ref			
	2017	285	136	(47.7)	1.2 [1.0–1.4]	0.11		
DR-TB treatment	MDR-TB	464	188	(40.5)	Ref		ref	
	XDR-TB	81	54	(66.7)	1.6 [1.4–2.0]	**<0.001**	1.5 [1.2–1.8]	**<0.001**
Age in years	≤30	87	16	(18.4)	ref		ref	
	31–40	142	61	(43.0)	2.3 [1.4–3.8]	**<0.001**	1.9 [1.2–3.1]	**0.006**
	41–50	150	70	(46.7)	2.5 [1.6–4.1]	**<0.001**	2.0 [1.2–3.2]	**0.004**
	>50	166	95	(57.2)	3.1 [2.0–4.9]	**<0.001**	2.2 [1.4–3.6]	**0.001**
Sex	Male	412	201	(48.8)	ref		ref	
	Female	133	41	(30.8)	0.6 [0.5–0.8]	**<0.001**	0.8 [0.6–1.1]	0.15
TB localization	Pulmonary	528	241	(45.6)	ref		ref	
	Extrapulmonary	17	1	(5.9)	0.1 [0.0–0.9]	**0.035**	0.2 [0.0–1.3]	0.09
Category of TB	New	152	47	(30.9)	ref		ref	
	Previously treated	393	195	(49.6)	1.6 [1.2–2.1]	**<0.001**	1.4 [1.1–1.7]	**0.02**
Cardiovascular	No/not reported	491	208	(42.4)	ref		ref	
	Yes	54	34	(63.0)	1.5 [1.2–1.9]	**<0.001**	1.2 [0.9–1.5]	0.13
Pulmonary	No/not reported	528	234	(44.3)	ref			
	Yes	17	8	(47.1)	1.1 [0.6–1.8]	0.82		
Gastrointestinal	No/not reported	512	227	(44.3)	ref			
	Yes	33	15	(45.4)	1.0 [0.7–1.5]	0.90		
Liver	No/not reported	501	210	(41.9)	ref		ref	
	Yes	44	32	(72.7)	1.7 [1.4–2.1]	**<0.001**	1.3 [1.0–1.7]	**0.04**
Anemia	No/not reported	511	231	(45.2)	Ref			
	Yes	34	11	(32.4)	0.7 [0.4–1.2]	0.19		
Diabetes mellitus	No/not reported	525	229	(43.6)	Ref		ref	
	Yes	20	13	(65.0)	1.5 [1.1–2.1]	**0.02**	1.4 [1.0–1.9]	**0.04**
HIV/AIDS	No/not reported	377	161	(42.7)	ref			
	Yes	168	81	(48.2)	1.1 [0.9–1.4]	0.22		
Smoking	No/not reported	278	110	(39.6)	ref		ref	
	Yes	267	132	(49.4)	1.2 [1.0–1.5]	**0.02**	1.0 [0.8–1.2]	0.73
Alcohol use	No/not reported	426	161	(37.8)	ref		ref	
	Yes	119	81	(68.1)	1.8 [1.5–2.1]	**<0.001**	1.5 [1.2–1.9]	**0.001**
Homeless	No/not reported	513	234	(45.6)	ref			
	Yes	32	8	(25.0)	0.5 [0.3–1.0]	0.052		
Former prisoner	No/not reported	517	228	(44.1)	ref			
	Yes	28	14	(50.0)	1.1 [0.8–1.7]	0.52		

Footnotes: TB = tuberculosis; MDR-TB = multidrug-resistant TB; XDR-TB = extensively drug-resistant TB; DR-TB = drug-resistant TB; RR = relative risk; aRR = adjusted relative risk; CI = confidence intervals. Bold shows statistical significance.

**Table 4:** Risk factors for death in persons with TB enrolled for MDR-TB and XDR-TB treatment in Tashkent city, Uzbekistan, 2016–2017.

Variables		Enrolled to Treatment	Death		RR (95% CI)	*p*-Value	aRR (95% CI)	*p*-Value
		n	n	(%)				
Total		545	117	(21.5)				
TB dispensary	City Tashkent	124	24	(19.4)	ref			
	Dispensary 1	44	12	(27.3)	1.4 [0.8–2.6]	0.26		
	Dispensary 2	87	22	(25.3)	1.3 [0.8–2.2]	0.30		
	Dispensary 3	85	19	(22.4)	1.2 [0.7–2.0]	0.60		
	Dispensary 4	57	9	(15.8)	0.8 [0.4–1.6]	0.57		
	Dispensary 5	148	31	(20.9)	1.1 [0.7–1.7]	0.74		
Year started treatment	2016	260	64	(24.6)	ref			
	2017	285	53	(18.6)	0.8 [0.5–1.0]	0.09		
DR-TB treatment	MDR-TB	464	88	(19.0)	ref		Ref	
	XDR-TB	81	29	(35.8)	1.9 [1.3–2.7]	**<0.001**	2.0 [1.4–3.0]	**<0.001**
Age in years	≤30	87	4	(4.6)	ref		Ref	
	31–40	142	30	(21.1)	4.6 [1.7–12.6]	**0.003**	3.5 [1.3–9.4]	**0.013**
	41–50	150	37	(24.7)	5.4 [2.0–14.5]	**0.001**	3.9 [1.5–10.4]	**0.006**
	>50	166	46	(27.7)	6.0 [2.2–16.2]	**<0.001**	3.8 [1.4–10.2]	**0.009**
Sex	Male	412	100	(24,3)	ref		Ref	
	Female	133	17	(12.8)	0.5 [0.3–0.8]	**0.008**	0.8 [0.5–1.2]	0.25
TB localization	Pulmonary	528	117	(22.2)	ref			
	Extrapulmonary	17	0	(0.0)	0.0 [0.0–0.0]	**<0.001**		
Category of TB	New	152	19	(12.5)	ref		Ref	
	Previously treated	393	98	(24.9)	2.0 [1.3–3.1]	**0.003**	1.6 [1.0–2.5]	**0.048**
Cardiovascular	No/not reported	491	93	(18.9)	ref		Ref	
	Yes	54	24	(44.4)	2.3 [1.7–3.3]	**<0.001**	2.2 [1.4–3.4]	**<0.001**
Pulmonary	No/not reported	528	112	(21.2)	ref			
	Yes	17	5	(29.4)	1.4 [0.7–2.9]	0.40		
Gastrointestinal	No/not reported	512	113	(22.1)	ref			
	Yes	33	4	(12.1)	0.5 [0.2–1.4]	0.21		
Liver	No/not reported	501	100	(20.0)	ref		ref	
	Yes	44	17	(38.6)	1.9 [1.3–2.9]	**0.002**	1.4 [0.9–2.3]	0.16
Anemia	No/not reported	511	113	(22.1)	ref			
	Yes	34	4	(11.8)	0.5 [0.2–1.4]	0.19		
Diabetes mellitus	No/not reported	525	111	(21.1)	ref			
	Yes	20	6	(30.0)	1.4 [0.7–2.8]	0.32		
HIV/AIDS	No/not reported	377	66	(17.5)	ref		ref	
	Yes	168	51	(30.4)	1.7 [1.3–2.4]	**0.001**	1.8 [1.2–2.7]	**0.002**
Smoking	No/not reported	278	46	(16.5)	ref		ref	
	Yes	267	71	(26.6)	1.6 [1.2–2.2]	**0.005**	1.2 [0.8–1.8]	0.46
Alcohol use	No/not reported	426	79	(18.5)	ref		ref	
	Yes	119	38	(31.9)	1.7 [1.2–2.4]	**0.001**	1.0 [0.7–1.5]	0.92
Homeless	No/not reported	513	114	(22.2)	ref			
	Yes	32	3	(9.4)	0.4 [0.1–1.3]	0.12		
Former prisoner	No/not reported	517	107	(20.7)	ref		ref	
	Yes	28	10	(35.7)	1.7 [1.0–2.9]	0.04	1.4 [0.8–2.5]	0.23

Footnotes: TB = tuberculosis; MDR-TB = multidrug-resistant TB; XDR-TB = extensively drug-resistant TB; DR-TB = drug resistant TB; RR = relative risk; aRR = adjusted relative risk; CI = confidence intervals. Bold shows statistical significance.

**Table 5:** Risk factors for loss to follow-up in patients with TB enrolled for MDR-TB and XDR-TB treatment in Tashkent city, Uzbekistan, 2016–2017.

Variables		Enrolled to Treatment	Lost-To-Follow-Up		RR (95% CI)	*p-*Value	aRR (95% CI)	*p*-Value
		n	N	(%)				
Total		545	32	(5.9)				
TB dispensary	City Tashkent	124	8	(6.5)	ref			
	Dispensary 1	44	2	(4.5)	0.7 [0.2–3.2]	0.65		
	Dispensary 2	87	7	(8.0)	1.2 [0.5–3.3]	0.66		
	Dispensary 3	85	5	(5.9)	0.9 [0.3–2.7]	0.87		
	Dispensary 4	57	3	(5.3)	0.8 [0.2–3.0]	0.76		
	Dispensary 5	148	7	(4.7)	0.7 [0.3–2.0]	0.54		
Year started treatment	2016	260	7	(2.7)	ref		ref	
	2017	285	25	(8.8)	3.3 [1.4–7.4]	**0.005**	4.9 [2.3–10.4]	**<0.001**
DR-TB treatment	MDR-TB	464	16	(3.4)	ref		ref	
	XDR-TB	81	16	(19.8)	5.7 [3.0–11.0]	**<0.001**	9.5 [5.1–17.5]	**<0.001**
Age in years	≤30	87	2	(2.3)	ref		ref	
	31–40	142	5	(3.5)	1.5 [0.3–7.7]	0.61	1.0 [0.2–4.8]	0.98
	41–50	150	11	(7.3)	3.2 [0.7–14.1]	0.12	2.5 [0.6–10.9]	0.24
	>50	166	14	(8.4)	3.7 [0.9–15.8]	0.08	2.1 [0.5–9.4]	0.31
Sex	Male	412	28	(6.8)	ref		ref	
	Female	133	4	(3.0)	0.4 [0.2–1.2]	0.12	1.0 [0.3–3.0]	0.99
TB localization	Pulmonary	528	32	(6.1)	ref			
	Extrapulmonary	17	0	(0.0)	0.0 [0.0–0.0]	**<0.001**		
Category of TB	New	152	5	(3.3)	ref			
	Previously treated	393	27	(6.9)	2.1 [0.8–5.3]	0.12		
Cardiovascular	No/not reported	491	30	(6.1)	ref			
	Yes	54	2	(3.7)	0.6 [0.1–2.5]	0.48		
Pulmonary	No/not reported	528	31‘	(5.9)	ref			
	Yes	17	1	(5.9)	1.0 [0.1–6.9]	0.99		
Gastrointestinal	No/not reported	512	31	(6.1)	ref			
	Yes	33	1	(3.0)	0.5 [0.1–3.6]	0.49		
Liver	No/not reported	501	30	(6.0)	ref			
	Yes	44	2	(4.5)	0.8 [0.2–3.1]	0.70		
Anemia	No/not reported	511	31	(6.1)	ref			
	Yes	34	1	(2.9)	0.5 [0.1–3.4]	0.47		
Diabetes mellitus	No/not reported	525	32	(6.1)	ref			
	Yes	20	0	(0.0)	0.0 [0.0–0.0]	**<0.001**		
HIV/AIDS	No/not reported	377	25	(6.6)	ref			
	Yes	168	7	(4.2)	0.6 [0.3–1.4]	0.27		
Smoking	No/not reported	278	17	(6.1)	ref			
	Yes	267	15	(5.6)	0.9 [0.5–1.8]	0.80		
Alcohol use	No/not reported	426	19	(4.5)	ref		ref	
	Yes	119	13	(10.9)	2.4 [1.2–4.8]	**0.009**	3.2 [1.6–6.3]	**0.001**
Homeless	No/not reported	513	32	(6.2)	ref			
	Yes	32	0	(0.0)	0.0 [0.0–0.0]	**<0.001**		
Former prisoner	No/not reported	517	31	(6.0)	ref			
	Yes	28	1	(3.6)	0.6 [0.1–4.2]	0.60		

Footnotes: TB = tuberculosis; MDR-TB = multidrug-resistant TB; XDR-TB = extensively drug-resistant TB; DR-TB = drug-resistant TB; RR = relative risk; aRR = adjusted relative risk; CI = confidence intervals. Bold shows statistical significance.

## Data Availability

The data that support the findings of this study are available from the corresponding author, K.S., upon reasonable request.
